# A Longitudinal Multilevel Study of the “Social” Genotype and Diversity of the Phenotype

**DOI:** 10.3389/fpsyg.2018.02034

**Published:** 2018-10-24

**Authors:** Elli Oksman, Tom Rosenström, Mirka Hintsanen, Laura Pulkki-Råback, Jorma Viikari, Terho Lehtimäki, Olli Tuomas Raitakari, Liisa Keltikangas-Järvinen

**Affiliations:** ^1^Department of Psychology and Logopedics, Faculty of Medicine, University of Helsinki, Helsinki, Finland; ^2^Unit of Psychology, Faculty of Education, University of Oulu, Oulu, Finland; ^3^Departments of Clinical Physiology and Nuclear Medicine, Turku University Hospital, Turku, Finland; ^4^Division of Medicine, Turku University Hospital, Turku, Finland; ^5^Research Centre of Applied and Preventive Cardiovascular Medicine, University of Turku, Turku, Finland; ^6^Department of Medicine, University of Turku, Turku, Finland; ^7^Fimlab Laboratories and Finnish Cardiovascular Research Center—Tampere, Department of Clinical Chemistry, Faculty of Medicine and Life Sciences, University of Tampere, Tampere, Finland

**Keywords:** personality assessments, personality development, longitudinal analysis, multilevel modeling, sociability, oxytocin gene

## Abstract

Sociability and social domain-related behaviors have been associated with better well-being and endogenous oxytocin levels. Inspection of the literature, however, reveals that the effects between sociability and health outcomes, or between sociability and genotype, are often weak or inconsistent. In the field of personality psychology, the social phenotype is often measured by error-prone assessments based on different theoretical frameworks, which can partly explain the inconsistency of the previous findings. In this study, we evaluated the generalizability of “sociability” measures by partitioning the population variance in adulthood sociability using five indicators from three personality inventories and assessed in two to four follow-ups over a 15-year period (*n* = 1,573 participants, 28,323 person-observations; age range 20–50 years). Furthermore, we tested whether this variance partition would shed more light to the inconsistencies surrounding the “social” genotype, by using four genetic variants (rs1042778, rs2254298, rs53576, rs3796863) previously associated with a wide range of human social functions. Based on our results, trait (between-individual) variance explained 23% of the variance in overall sociability, differences between sociability indicators explained 41%, state (within-individual) variance explained 5% and measurement errors explained 32%. The genotype was associated only with the sociability indicator variance, suggesting it has specific effects on sentimentality and emotional sharing instead of reflecting general sociability.

## Introduction

The explosive growth of use and influence of social media in the society has increased the pressure to understand “sociability” at the level of basic research (Ross et al., 2009; Correa et al., 2010; Hughes et al., 2012). Every personality theory includes the concept of sociability in some form. The social phenotype is among the features that are always evaluated when an individual's personality is being measured. According to one well-established definition, “sociability” refers to the tendency to seek the presence of others and be fond of the company of others, which has been shown to be biologically important for highly social species (Buss, 1991; Réale et al., 2007; Caldwell, 2012). In primates, the tendency for social behavior has several fitness consequences, such as better ability to survive in stressful situations, longer life expectancy, and a greater number of offspring (Silk et al., 2003, 2010; Silk, 2007; Dunbar and Shultz, 2010). Similarly, in humans higher sociability has been associated with better physical health (Cohen et al., 2003), lower environment-related stress sensitivity (Swickert et al., 2002; Hintsanen et al., 2011), and a higher rate of childbearing (Jokela et al., 2009), but also with a lower risk for developing clinical symptoms such as depression and anxiety (Malouff et al., 2005; Clements and Bailey, 2010; Cloninger et al., 2010; Elovainio et al., 2015). Whether a high or low level of sociability is more adaptive for the individual may depend on the environment (Friedman, 2000; Cote et al., 2008) and, to some extent, on gender (Silk, 2007; Pachucki et al., 2014). On the whole, however, high sociability and social support have more often been associated with favorable than with harmful outcomes.

Building on animal research, the genetic background of human's social behavior, social cognition and prosociality—particularly, that related to endogenous oxytocin levels—has witnessed a surge of interest during the past decade (e.g., Ross and Young, 2009; Caldwell, 2012; Feldman et al., 2016). A wide array of studies has shown that individual differences in endogenous oxytocin levels, polymorphisms of the oxytocin receptor (OXTR) gene and even exogenously administered (e.g., intranasal) oxytocin may influence a range of outcomes related to the social domain (Table 1). Furthermore, some of the results have generated excitement about the potential implications for the current development of novel clinical approaches for mental disorders associated with social deficits (e.g., autism spectrum disorder, social anxiety disorder and borderline personality disorder; Heinrichs et al., 2009). Inspection of the literature, however, reveals that different assessments of social behavior make it difficult to directly compare the effects of oxytocin on the social phenotypes (Table 1). Furthermore, the error-prone assessments for sociability are often weak or inconsistent (Bartz et al., 2011; Bakermans-Kranenburg and van IJzendoorn, 2014; Dick et al., 2015). Based on the critical discussion about the consistency of personality traits that prevailed in the 1960's (Mischel, 1968; Mischel and Shoda, 1995), an interactionist approach has been widely used to clarify the role of oxytocin in human sociability and social behavior (Bartz et al., 2011). More recently, however, gene-environment interaction research has been challenged on several frequently overlooked grounds, such as their low power, small sample sizes and failure to replicate original findings (Dick et al., 2015).

**Table 1:** Examples of inconsistencies in the prior findings between sociability, or social behavior closely related to different aspects of sociability, and “social” genotype.

**Study**	**Type**	**Social phenotype**	**Social genotype**	**Conclusions**
Bakermans-Kranenburg and van IJzendoorn, 2014	Meta-analysis	Personality, social behavior	OXTR rs53576 OXTR rs2254298	SNPs failed to explain a statistically significant part of human social behavior.
Bartz et al., 2011	Review	Social cognition, prosociality	Exogenous oxytocin	No main effect in 43% of the studies; conditional positive effect (e.g., situational or individual differences) in 63%; negative effect in 21%.
Brüne, 2012	Review	Sociability, risk for psychiatric disorders (e.g., autism, social anxiety)	OXTR rs2254298	If the genotype is associated with early environmental adversities, it may results in developing psychopathologies (e.g., autism); under thriving conditions, it may have advantageous effects on an individual's social network.
Caldwell, 2012	Review	Sociability (in animals and in humans)	AVP OXT	Results on the role of OXT and AVP in the regulations of sociability across species are promising.
Cataldo et al., 2018	Review	Social and affiliative behaviors, Autism Spectrum Disorder (ASD)	AVP OXT	Although the genes did not surface in genome-wide association studies, evidence supported the hypothesis that these receptors are widely involved in the regulation of social behavior and contribute to the etiology of ASD.
Feldman et al., 2016	Review	Affiliation, sociality, social relationships	OXTR rs7632287 OXTR rs1042778 OXTR rs2268494 OXTR rs2268490 OXT rs2740210 OXT rs4813627 OXT rs4813625 CD38 rs3796863 CD38 rs6449197	Studies provide evidence for the involvement of OT-pathway genes in human social functions. However, factors such as gender, culture, and early environment often confound attempts to replicate first findings.
Harari-Dahan and Bernstein, 2014	Review	Social behavior, social approach and avoidance motivation	OXT	Social and non-social effects of OXT may be mediated by social approach-avoidance motivation processes.
Heinrichs et al., 2009	Review	Social behavior, social cognition	AVP OXT	OXT associated with responses to socially relevant challenges; with responses to positive social interactions; with amygdala reactivity to social stimuli; with social cognition; and with several mental disorders characterized by social difficulties (e.g., autism). AVP may influence social communication, but in a sex-specific manner.
Li et al., 2015	Meta-analysis	General sociality, close relationships	OXTR rs53576	GG allele associated with higher general sociality than AA/AG allele carriers, but no association was found between close relationships and rs53576.
Tops et al., 2018	Review	Social behavior, Autism Spectrum Disorder (ASD)	OXT	SNPs in the OXTR gene are linked to deficits in social behavior and ASD, but with no consensus on which SNPs are associated with pro- and antisocial behavior.
Torres et al., 2018	Review	Socio-emotional development, social behavior, sociability	AVP OXT	AVP and OXT may contribute to different dimensions of normal and pathological socio-affective functioning. Differences between life stages may exist.

*SNP, Single nucleotide polymorphisms; OXTR, the oxytocin receptor; OXT, the structural gene for oxytocin; AVP, the arginine vasopressin receptor*.

The inconsistency of the results regarding the genes underlying sociability may be due differences in research methods or due to statistical issues, but also due fundamental differences in the definition of human sociability (e.g., is the focus on behavioral, motivational or affective aspects of sociability). In other words, it is not entirely clear how comparable the meaning of the term “sociability” is across different studies, how sociability develops in general and how the distinct indicators of sociability develop over the adult lifespan. With animals, sociability has usually been studied by observing the actual number and quality of an individual's social bonds and the group dynamics as a whole (e.g., grooming and mating behavior; Silk, 2007; Dunbar, 2010). With humans, sociability measures mostly rely on self-reports because of their practicality and because they reflect the person's self-concept (Robins et al., 2007), though there are some exceptions (e.g., by observing and making predictions based on one person's behavioral consistency in different situational contexts; Mischel, 2004; Kammrath et al., 2005). However, self-report questionnaires are often grounded in different theories or pragmatic solutions, and the overt indices, as well as the underlying construct of sociability, can, therefore differ from one study to another (Lucas et al., 2000). In other words, there is a fundamental problem regarding the incoherence of self-evaluated social phenotype. While some different indicators of sociability may have been previously compared against each other, and some differences probably always emerge in such comparisons, those differences can be difficult to place in a wider context of social interaction without reference points like the use of the individual's past and future as a control, or without the use of other widely-used measures of sociability.

Studying the longitudinal variation in different indicators of sociability would move the literature on human sociability into a wider empirical context and give insight into the extent to which findings on adult sociability can be generalized from one individual, inventory, time-point or gender to another. Crucially, studying multiple levels of the variance in sociability in a single study could reveal explanations for the inconsistencies in past genetic findings, if the genotypes affect some levels but not others. Furthermore, when the environment is more variable than the phenotype, a longitudinal within-individual variance component can indirectly reveal a gene-by-environment interaction (GxE), while circumventing many of the known statistical issues in GxE studies (e.g., low power and failure to replicate original findings; Dick et al., 2015). In other words, if a time-constant genotype increases effects of a fast-varying environment (e.g., life events) on a slowly varying phenotype (e.g., personality), the genotype will necessarily increase within-individual variance in the phenotype. This can be tested without direct access to the life events.

In the present study, we have to aims: first, to provide a well-powered quantitative estimate of the extent to which the population variance in sociability can be attributed to trait (between-individual) variance, to differences among commonly used inventories, to state variance (within-individual changes over time) or measurement error, and second, to assess the effect of oxytocin genes on that multilevel variance partition. To this end, we examined five different indicators of adulthood sociability derived from three commonly used personality inventories, the Neuroticism-Extraversion-Openness Five-Factor Inventory (NEO-FFI), the Temperament and Character Inventory (TCI) and the Emotionality-Activity-Sociability (EAS) Temperament Survey (hereafter referred to as the “EAS”). These indicators were assessed over a 15-year follow-up period in a representative, population-based sample covering the age range of 20 to 50 years. Additionally, genomic DNA was extracted from peripheral blood leukocytes (Raitakari et al., 2008).

We deliberately chose to use these five sociability indicators in the present study as they focus on different aspect of “social” or “extraverted” preferences and tendencies. This diversity enabled us to study overlap between seemingly distinct measures across time (possibly tapping into the core of sociability). Thus, this study provides a reference on the generalizability of common indicators of social behavior. In addition, the model we present offers principled ways to study how the development of sociability (within-individual variance) is differentially susceptible to certain genotypes, and how these genotypes relate to the between-individual differences or different indicators of sociability. In comparison to direct GxE research, this approach will instead inform how differences in genetic influences on developing sociability might be reflected in within- or between-individual variance of sociability, or in variance between the sociability indicators.

## Materials and methods

### Participants

The participants were derived from the ongoing longitudinal population-based Young Finns Study (Viikari et al., 1982; Raitakari et al., 2008). The original sample of 3,596 subjects (of which 1,832, or 51%, were girls) was selected in 1980 from five Finnish university cities with a medical school and their surrounding suburban and rural areas as a representative sample of the Finnish population. The subjects were healthy children and adolescents randomly selected from six age-based cohorts (those born in 1962, in 1965, in 1968, in 1971, in 1974 and in 1977) which have now been followed for 32 years in eight study waves done in years 1983, 1986, 1989, 1992, 1997, 2001, 2007, and 2010–2012. The study was approved by the local ethics committee of the Finnish Advisory Board on Research Integrity (TENK), it is in accordance with Declaration of Helsinki, and the participants and their parents gave written consent.

In this study, we focus on the assessment waves of 1997 (age range 20 to 35), 2001, 2007 and 2012 (age range 35 to 50), covering participants in an age range between 20 to 50 years (*N* = 1 978, of which 1 165, or 59%, were women). We selected these study waves because four of the sociability indicators were assessed four times and one of them twice during this time period, and the assessments represent the participants at the transition from young adulthood to late adulthood. The number of participants with both data on social phenotype and the genotype was 1,573. This forms the total sample used in the present study. To our knowledge, the present aim has not been previously explored, and therefore we did not have a pre-specified effect size. However, the sample size should be sufficient to accurately distinguish even small differences in the studied variance components, as is evident from our confidence interval estimates.

### Personality questionnaires

Adulthood sociability was assessed by self-reports on the following: (1) Extraversion scale from the NEO-FFI (McCrae and Costa, 1988; Costa and McCrae, 1992), (2) the three Reward Dependence subscales derived from the TCI (Cloninger, 1987; Cloninger et al., 1993) and (3) the Sociability scale from the EAS (Buss and Plomin, 1975, 1986). In the Young Finns Study, the NEO-FFI has so far been administered twice, in 2007 and 2012, and the TCI and the EAS have been administered four times, in 1997, 2001, 2007, and 2012. With all these inventories, for each item a five-point scale ranging from “1: Definitely false” to “5: Definitely true” was used from 1997 to 2001. From 2007 onwards, the response options were slightly modified to have a range from “1: [The definition fits me] poorly or not at all” to “5: [The definition fits me] very well.” The mean score for the used sociability indicator was calculated for the participants who answered at least 75% of the trait items. All the sociability indicators correlated with each other (*r* = 0.12–0.66, *p* < 0.001). The Cronbach's alphas for each indicator are presented in Table 2. Based on skewness and excess-kurtosis estimates (ranging from −0.47 to −0.02 and from −0.39 to 0.00, respectively) and graphical analyses, the data characteristics closely corresponded to the normality assumption.

**Table 2:** Descriptive statistics for the five indicators of adulthood sociability by assessment wave and the prevalence of genotype in the Young Finns Study (*N* = 3 596).

	**Total sample**			**Women**		**Men**	
	***Mean (SD) or %***	***N***	**α**	***Mean (SD) or %***	***n***	***Mean (SD) or %***	***n***
**NEO-FFI**							
Extraversion							
2007	3.39 (0.55)	2051	0.81	3.43 (0.55)	1209	3.32 (0.55)	842
2012	3.37 (0.57)	1736	0.83	3.43 (0.57)	1021	3.29 (0.57)	715
**TCI**
Sentimentality							
1997	3.16 (0.54)	2106	0.69	3.32 (0.50)	1264	2.91 (0.51)	842
2001	3.12 (0.54)	2100	0.69	3.30 (0.49)	1215	2.87 (0.51)	885
2007	3.05 (0.54)	2056	0.73	3.22 (0.50)	1211	2.81 (0.51)	845
2012	3.04 (0.55)	1745	0.73	3.20 (0.51)	1023	2.81 (0.52)	722
Social attachment							
1997	3.63 (0.73)	2106	0.82	3.82 (0.71)	1264	3.36 (.68)	842
2001	3.66 (0.74)	2102	0.83	3.86 (0.71)	1216	3.37 (0.69)	886
2007	3.57 (0.74)	2056	0.83	3.76 (0.67)	1211	3.30 (0.67)	845
2012	3.54 (0.71)	1743	0.82	3.73 (0.68)	1023	3.27 (0.66)	720
Dependence							
1997	3.25 (0.54)	2106	0.53	3.28 (0.52)	1264	3.20 (0.56)	842
2001	3.32 (0.55)	2098	0.57	3.38 (0.55)	1215	3.23 (0.55)	883
2007	3.37 (0.54)	2056	0.60	3.45 (0.53)	1211	3.25 (0.53)	845
2012	3.35 (0.53)	1744	0.59	3.43 (0.52)	1023	3.25 (0.53)	721
**EAS**							
Sociability							
1997	3.46 (0.76)	2103	0.79	3.57 (0.77)	1263	3.29 (0.72)	840
2001	3.39 (0.74)	2105	0.78	3.50 (0.75)	1216	3.25 (0.71)	889
2007	3.27 (0.72)	2056	0.79	3.37 (0.72)	1210	3.14 (0.68)	846
2012	3.25 (0.72)	1751	0.80	3.33 (0.74)	1025	3.14 (0.69)	726
**Genotype**							
OXTR rs1042778							
**TT**	15.4 %	360	–	15.2 %	193	15.5 %	167
GG/GT	84.7 %	1985	–	84.8 %	1074	84.5 %	911
OXTR rs2254298							
**GG**	84.3 %	1976	–	84.8 %	1074	83.7 %	902
AA/AG	15.7 %	369	–	15.2 %	193	16.3 %	176
OXTR rs53576							
**AA/AG**	66.4 %	1557	–	66.1 %	837	66.8 %	720
GG	33.6 %	788	–	33.9 %	430	33.2 %	358
CD38 rs3796863							
**CC**	40.8 %	957	–	41.5 %	526	40.00 %	431
AA/AC	59.2 %	1388	–	58.5 %	741	60.0 %	647
Genetic risk score							
0	2.6 %	61	–	2.6 %	33	2.6 %	28
1	27.5 %	645	–	28.3 %	358	26.6 %	287
2	46.4 %	1089	–	45.9 %	582	47.0 %	507
3	21.0 %	493	–	20.5 %	260	21.6 %	233
4	2.4 %	57	–	2.7 %	34	2.1 %	23

*α, Cronbach's alpha; NEO-FFI, The Neuroticism-Extraversion-Openness Five-Factor Inventory; TCI, Temperament and Character Inventory; EAS, Emotionality-Activity-Sociability Temperament Survey. The bolded genotype represent the alleles that have been associated with risk of social difficulties. The genetic risk score was computed by summing the number of these genetic risk variations*.

#### The NEO-FFI

NEO-FFI was originally developed to provide a concise measure of the so-called Big Five basic personality factors (namely, Openness, Conscientiousness, Extraversion, Agreeableness, and Neuroticism; McCrae and Costa, 2004). Extraversion describes the warmth and gregariousness of a person (e.g., “I really like to discuss with people”), but also tendencies for assertiveness, seeking leadership or power, being active, excitement seeking and experiencing positive emotions. According to Five Factor theory, Extraversion can be divided into six subscales focusing on these different elements (namely, Warmth, Gregariousness, Assertiveness, Activity, Excitement Seeking, and Positive Emotions). However, in the short version of NEO-FFI used in the Young Finns Study, Extraversion is measured with 12 items in total, meaning only two items for each subscale; thus, we preferred to use the full scale.

#### The TCI

The TCI is based on the psychobiological theory of personality, including four dimensions of temperament and three dimensions of character (Cloninger, 1987; Cloninger et al., 1993). The temperament dimensions (namely, Novelty Seeking, Harm Avoidance, Reward Dependence, and Persistence) are associated with facets of behavioral conditioning; of these, Reward Dependence is related to “sociability” and social behavior tendencies (Cloninger et al., 1993). It measures the person's willingness to maintain behavior that has been previously rewarded, for example, by recognition and respect from others.

Reward Dependence is divided into three subscales—Sentimentality, Social Attachment, and Dependence—that are used separately in this study. Sentimentality (comprised of 10 items) measures a tendency to be deeply moved by sentimental appeals and the inclination to show, share and adapt emotions easily in the presence of others (e.g., “I like to please other people as much as I can”). People who score low on this subscale are described as practical and less sensitive to others' feelings. Social Attachment (eight items) measures a person's tendency to prefer company and intimacy over solitude and privacy (e.g., “I would like to have warm and close friends with me most of the time”). Dependence (six items) measures a person's need for emotional support and approval from others, combined with a tendency to please and be preoccupied with fears of being abandoned (e.g., disagrees with statements like “I don't care very much whether other people like me or the way I do things”). With this subscale, all six items were presented in a reversed format so that disagreeing with the claim indicates higher Dependence. The calculated score for the scale was converted to make it comparable with the scores for the other scales.

#### The EAS

The EAS was originally developed based on the notion that emotionality, activity, and sociability form the essential foundation of individuality in the sense that they appear early in development, are relatively stable and are proposed to be among the most heritable traits in personality as indicated by molecular behavioral genetic research (Buss and Plomin, 1975; Buss, 1991). The EAS Sociability scale assesses a tendency to prefer and enjoy the presence of others over being alone, and how comfortable a person feels in a group (e.g., “I like to be with people”). People who score high on the scale have a strong tendency to seek the company of others and prefer a great number of friends because of the social interactions' intrinsic rewards, such as sharing an activity or getting attention from others.

### The “social” genotype

The genetic variables have been frequently used in previous literature and can be considered as fixed-effect covariates in our statistical models. Specifically, variation over 670 000 genome-wide single nucleotide polymorphism (SNP) analyses (SWAS) was measured in total from 2,442 participants of the Young Finns Study in 2009. In the present study, we focused on genetic variants rs1042778, rs2254298, and rs53576 from oxytocin receptor gene (OXTR) which have been previously associated with social behavior, attention to social cues and formation of intimate, close relationships, and on rs3796863 from CD38 gene which has shown to moderate plasma oxytocin level (Tost et al., 2010; Feldman et al., 2012). Namely, we focused on OXTR rs1042778 TT, rs2254298 GG, rs53576 AA/AG, and CD38 rs3796863 CC alleles (Table 2) which have been recognized as a risk factors for variety of social difficulties, such as greater risk for autism and lower empathy (e.g., Bakermans-Kranenburg and van IJzendoorn, 2014; Feldman et al., 2014). The literature has demonstrated that cumulative effects of genes on a given phenotype, computed by combining several SNPs, tend to provide a better risk estimate for the outcome than each SNP alone (Belsky et al., 2009; Belsky and Israel, 2014; Feldman et al., 2014). Thus, in the first instance, we tested the effect of genetic risk score on the variance partitioning and social phenotype, and additionally, as a supplementary analysis, the effects of individual SNPs. A cumulative genetic risk score was computed by summing the number of genetic risk variations. The risk score ranged from 0 (no risk) to 4 (risk on all four SNPs).

### Statistical analyses

We partitioned the variance in the sociability indicators using multilevel, or mixed-effects, modeling, as the method recognizes the hierarchical structure of the data (Gelman and Hill, 2007; Dingemanse and Dochtermann, 2012). A mixed-effect model always includes at least one typical regression-model intercept (a “fixed” effect), plus one or more “random” effects. A random effect can be, for example, an individual-specific intercept when each has multiple longitudinal measurements. It is called a “random effect” because the multiple intercepts are not explicitly estimated, but assumed to be normally distributed; then only the magnitude of this variance component is estimated, that is, the variance of the latent intercepts. The variance of individual-specific intercepts corresponds to the variance due to stable between-individual differences, being closely related to the concept of personality when applied to behavior (Dingemanse and Dochtermann, 2012). Whether or not such between-individual differences are pertinent to a wider concept of sociability or just to specific indicators remains unknown without having either a gold-standard measure or the ability to estimate a variance component for the indicators.

In our models, the (unobserved) random effects can be represented by the following formula:

(1) $$\begin{array}{r} y_{i}\text{=}\gamma_{k\left[i\right]}+\alpha_{j\left[i\right]}+\delta_{t\left[i\right]}+\varepsilon_{i} \end{array}$$

where *y*_*i*_ corresponds to an observation *i* of overall adulthood sociability, as assessed by any of the indicators. Regarding the random effects that introduce variability to overall sociability, γ_*k*[*i*]_ refers to an intercept that is time- and questionnaire-constant, but individual-specific and thus its estimated variance component indicates the overall trait (between-individual) variance in the data [*k*[*i*] referring to the individual for which *y*_*i*_ was observed]. In contrast, α_*j*[*i*]_ refers to a model intercept that varies across both individuals and the sociability indicators but is fixed across time; thus, its estimated variance component captures the indicator variance in the sample, given that a latent intercept for an individual's average already exists [*j*[*i*] refers to the indicator and individual for which the value *y*_*i*_ was observed]. By similar logic, δ_*t*[*i*]_ stands for an individual- and time-specific intercept that is constant across the sociability indicators; because we already had an intercept for the individual, and an intercept for the sociability indicator, the variance of this intercept captures that of the within-individual changes (state variance) over time [*t*[*i*] referring to the individual and the follow-up in which *y*_*i*_ was observed]. Finally, ε_*i*_ refers to the error variance, including the variance that cannot be attributed to an individual, to follow up or to a sociability indicator. This error variance includes both measurement errors in individual indicators as well as within-individual changes that are not consistent across the indicators (i.e., do not reflect overall sociability nor stable indicator-specific differences). In our models, random-intercepts that define the correlation structure were not allowed to correlate. For more technical notations, see Supplementary Material 1 on the model equations and random-effects design matrix.

Finally, a fixed variable, such as genotype, could affect a random effect. For example, δ_*t*[*i*]_ might decompose as δ_*t*[*i*]_ = δ_*t*[*i*]_^*^ + ξ_*t*[*i*]_*x*_*i*_, where another random effect ξ_*t*[*i*]_ increases variance for one genotype (*x*_*i*_ = 1) relative to another (*x*_*i*_ = 0). We also test such interactions with genotype. Specifically, the interaction between genotype and within-individual variance is of high interest for researchers interested in GxE interactions, because genetic sensitivity to an environment is expected to increase within-individual variation.

The distribution of observations by age range and assessment wave are presented in Table 3. To standardize the overall sociability score into the same scale, we used the year 2007 as a reference time-point for each measure because it was the first year when all the sociability indicators were administered (every indicator had a population mean of zero and variance of one in 2007, but not necessarily at the other follow-ups).

**Table 3:** A number of distinct person-observations (*n*_*y*(*i*)_) at each assessment wave, by participant age, in the Young Finns Study.

	**Age range (years)**										
Wave	20–22	23–25	26–28	29–31	32–34	35–37	38–40	41–43	44–47	48–50	Σ
1997	1,452	1,556	1,616	1,636	1,640	1,480	–	–	–	–	9,380
2001	–	1,452	1,556	1,616	1,636	1,640	1,480	–	–	–	9,380
2007	–	–	–	1,815	1,945	2,020	2,045	2,050	1,850	–	11,725
2012	–	–	–	–	–	1,815	1,945	2,020	4,095	1,850	11,725
Σ	1,452	3,008	3,172	5,067	5,221	6,955	5,470	4,070	5,954	1,850	42 210

#### Fixed-effect covariates

First, we controlled for the assessment wave, and participants' gender, age and birth cohort in the model, entered as fixed-effects covariates. The earliest assessment wave (1997) were set as 0 and others got a value based on the number of years since 1997. However, as there were no birth cohort effects, and because this model and the model without birth cohort did not differ significantly, we preferred the model with fewer number of parameters and smaller Akaike's information criterion (AIC); that is, the model with assessment wave, and participant's gender and age as fixed-effects. Secondly, because women scored higher than men on almost all indicators of sociability at every measurement time (*p* < 0.001), except a non-significant gender difference for Extraversion, we additionally tested whether the variance partition differed between genders. Thirdly, we assessed the effect of genotype related to oxytocin pathway genes (i.e., “social” genotype”) on the multilevel variance partition. The R code for the performed analyses are presented in Supplementary Material 2.

#### Software

We conducted all statistical analyses using R software version 3.3.2., supplemented with the “lme4” package, version 1.1–7 (Bates et al., 2015). For figures demonstrating the developmental trends of sociability, we used a local polynomial regression fitting (loss) from the ggplot2 package, version 1.0.1 (Wickham, 2009) for weighted curve smoothing with the default control parameters.

## Results

### The variance partition of adulthood sociability

**Figure 1:**
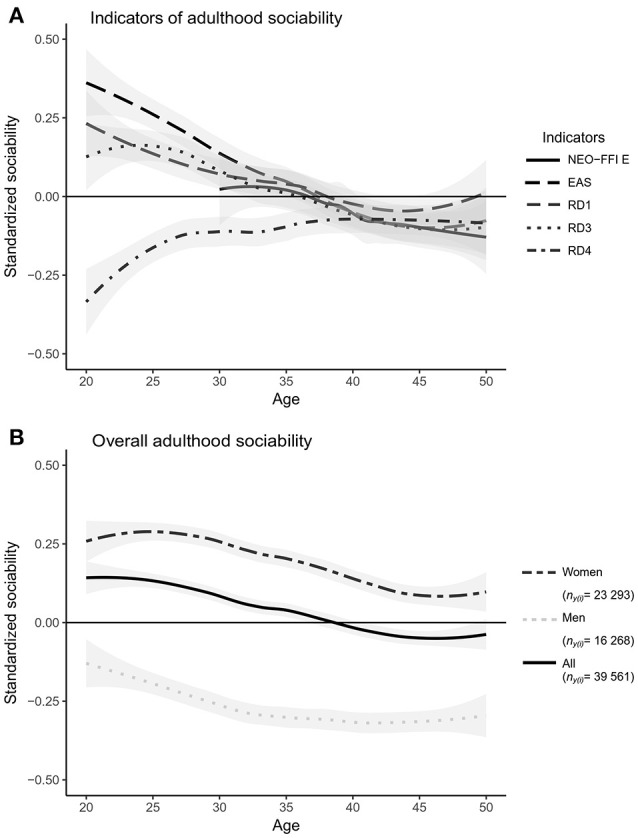
Differences in indicators of adulthood sociability **(A)**, and developmental trends in standardized overall sociability, as assessed by any of the five indicators used in the present study **(B)**. The trends are local polynomial regression fits, not from a multilevel model. NEO-FFI E, NEO-FFI Extraversion scale; EAS, EAS Sociability scale; RD1, TCI RD1: Sentimentality scale; RD3, TCI RD3: Social Attachment scale; RD4, TCI RD4: Dependence scale. All the sociability indicators were standardized to have a population mean of zero and variance of one in 2007. The gray area represents the 95% confidence interval.

The first aim of the present study was to compare coherence and stability of five self-reported indicators for social phenotype with a well-powered quantitative estimates of variance components for overall adulthood sociability. According to our results, the trait (between-individual) variance was 0.212 (95% CI = 0.192 to 0.233), the sociability indicator variance was 0.387 (CI = 0.371 to 0.403), the state (within-individual) variance was 0.048 (CI = 0.043 to 0.053) and the residual variance was 0.298 (CI = 0.291 to 0.305). In other words, the breakdown of variance in overall adulthood sociability was as follows: trait variance accounted for 22% of the variance, differences between the indicators accounted for 41%, age-related and individual-specific changes that were homogeneous across the different questionnaire measures accounted for 5%, and entirely idiosyncratic differences and/or measurement errors accounted for 32% of the variance (see Model 1 in Table 4). In these analyses, the model was only controlled for participants' age and gender. Differences between sociability indicators were larger during the age span of 20 to 30 years, whereas after age 35 the differences decreased (Figure 1A). However, in our sample, the number of people who were 20 to 30 years old during the follow-ups was smaller than the number of people who were 30 to 40 years old (Table 3). In addition to the indicator variance, trait (between-individual) variance explained ~4 times more of the total variance in adulthood sociability than did state (within-individual) variance (Table 4). In other words, the results indicate that, with respect to self-evaluated overall sociability, people differ more from each other than they differ from themselves from one time point to another.

**Table 4:** Multilevel model predicting standardized overall adulthood sociability (*n* = 1 573 participants, *n*_*y*(*i*)_ = 28 323 person-observations).

	**Fixed effects:**			**Random effects:**		
**Indicators of overall sociability**	**Estimate**	**SE**	***P*-value**	**Var**	***SD***	**Var%**
**Model 1**						
(Intercept)	0.438	0.071	< 0.001	–	–	–
Age	−0.007	0.003	0.006	–	–	–
Gender (0 = women, 1 = men)	−0.442	0.025	< 0.001	–	–	–
Assessment wave	−0.001	0.003	0.586	–	–	–
Overall between–individual variance (*γ_*k*[*i*]_*)	–	–	–	0.212	0.461	22.5
Sociability indicator variance (*α_*j*[*i*]_*)	–	–	–	0.387	0.622	40.9
Within individual change over time (*δ_*t*[*i*]_*)	–	–	–	0.048	0.219	5.1
Residual (*ε_*i*_*)	–	–	–	0.298	0.546	31.5
**Model 2**						
(Intercept)	0.470	0.077	< 0.001	–	–	–
Age	−0.007	0.003	0.005	–	–	–
Gender (0 = women, 1 = men)	−0.441	0.025	< 0.001	–	–	–
Genetic risk score	−0.016	0.015	0.304	–	–	–
Assessment wave	−0.001	0.003	0.597	–	–	–
Overall between-individual variance (*γ_*k*[*i*]_*)	–	–	–	0.212	0.461	22.8
Sociability indicator variance (*α_*j*[*i*]_*)	–	–	–	0.370	0.608	39.7
Within individual change over time (*δ_*t*[*i*]_*)	–	–	–	0.048	0.219	5.2
Residual (*ε_*i*_*)	–	–	–	0.298	0.546	32.0
**GxE interactions**	–	–	–			
G x *γ_*k*[*i*]_*	–	–	–	0.000	0.000	0.0
G x *α_*j*[*i*]_*	–	–	–	0.004	0.061	0.3
G x *δ_*t*[*i*]_*	–	–	–	0.000	0.000	0.0

*Model 1: Five sociability indicators represent—overall adulthood sociability*.

*Model 2: Genetic risk score and GxE interactions included to Model 1. “G” corresponds to the fixed-effect genetic risk score, but its interaction is taken with the random-effects (i.e., they are multiplied)*.

Of the fixed effects, both gender (β = −0.442; SE = 0.025; *P* < 0.001) and age (β = −0.007; SE = 0.003; *P* = 0.006) of a participant predicted differences in overall adulthood sociability. Men had lower overall sociability than did women, and in both genders, overall sociability decreased with age (Figure 1B). When we analyzed data for men and women separately, the variance partitioning remained similar; however, age explained total adulthood sociability only for women (β = −0.008; SE = 0.003; *P* = 0.017).

### The “social” genotype

Our second aim was to assess the effect of oxytocin genes on the multilevel variance. The genotype did not associate with overall adulthood sociability as a fixed-effects covariate (Model 2 in Table 4). Regarding the variance components, including the genetic information in the model changed the sociability indicator variance which decreased to 0.370 (CI = 0.391 to 0.446). The other variance components did not change notably after the inclusion of genetic information. In other words, after including the genotype to the model, the differences between the sociability indicators accounted for 40% of the total variance in sociability (1% less than without the genotype). Accordingly, we found the only notable interaction effect between the genetic risk score and any of the random-effect variances for the sociability indicators variance. The interaction effect explained ~0.3% of the variance in overall sociability (Table 4). With individual SNPs, the interaction effect between the genotype and sociability indicators was slightly stronger with OXTR rs1042778 TT, rs2254298 GG, and CD38 rs3796863 CC alleles which explained 1.6, 5.5, and 1.4% of the variance in overall adulthood sociability, respectively (Table 5). In other words, in comparison with the genetic risk score, especially rs2254298 GG associated differentially with the overall adulthood sociability. However, when we analyzed SNPs and sociability indicators separately, the strongest associations were found for rs1042778 TT and rs379663 CC alleles instead of rs2254298 GG. These two SNPs associated with the kind of sociability emphasizing sentimentality and sharing emotions with others (i.e., TCI RD1: Sentimentality), but not with the other forms of sociability (Table 6).

**Table 5:** Individual SNPs and GxE interactions predicting standardized overall adulthood sociability (*n* = 1 573 participants, *n*_*y*(*i*)_ = 28 323 person-observations).

	**Fixed effects:**			**Random effects:**		
**Indicators of overall sociability**	**Estimate**	**SE**	***P*-value**	**Var**	***SD***	**Var %**
**Model 1—OXTR rs3796863**						
(Intercept)	0.444	0.073	< 0.001	–	–	–
Age	−0.007	0.003	0.006	–	–	–
Gender (0 = women, 1 = men)	−0.441	0.025	< 0.001	–	–	–
rs3796863 TT-allele	−0.010	0.026	0.701	–	–	–
Assessment wave	−0.001	0.003	0.583	–	–	–
Overall between-individual variance (*γ_*k*[*i*]_*)	–	–	–	0.212	0.460	22.3
Sociability indicator variance (*α_*j*[*i*]_*)	–	–	–	0.377	0.614	39.7
Within individual change over time (*δ_*t*[*i*]_*)	–	–	–	0.048	0.219	5.1
Residual (*ε_*i*_*)	–	–	–	0.298	0.546	31.3
**GxE interactions**						
G x *γ_*k*[*i*]_*	–	–	–	0.000	0.000	0.0
G x *α_*j*[*i*]_*	–	–	–	0.015	0.124	1.6
G x *δ_*t*[*i*]_*	–	–	–	0.000	0.000	0.0
**Model 2—OXTR rs2254298**						
(Intercept)	0.441	0.071	< 0.001	–	–	–
Age	−0.007	0.003	0.006	–	–	–
Gender (0 = women, 1 = men)	−0.442	0.025	< 0.001	–	–	–
rs2254298 GG-allele	−0.018	0.035	0.613	–	–	–
Assessment wave	−0.001	0.003	0.580	–	–	–
Overall between-individual variance (*γ_*k*[*i*]_*)	–	–	–	0.212	0.460	21.3
Sociability indicator variance (*α_*j*[*i*]_*)	–	–	–	0.378	0.614	37.9
Within individual change over time (*δ_*t*[*i*]_*)	–	–	–	0.047	0.216	4.7
Residual (*ε_*i*_*)	–	–	–	0.298	0.546	29.9
**GxE interactions**						
G x *γ_*k*[*i*]_*	–	–	–	0.000	0.000	0.0
G x *α_*j*[*i*]_*	–	–	–	0.055	0.234	5.5
G x *δ_*t*[*i*]_*	–	–	–	0.007	0.086	0.7
**Model 3—OXTR rs53576**						
(Intercept)	0.442	0.072	< 0.001	–	–	–
Age	−0.007	0.003	0.005	–	–	–
Gender (0 = women, 1 = men)	−0.441	0.025	< 0.001	–	–	–
rs53576 AA/AG-alleles	−0.010	0.027	0.710	–	–	–
Assessment wave	−0.001	0.003	0.587	–	–	–
Overall between-individual variance (*γ_*k*[*i*]_*)	–	–	–	0.212	0.460	22.4
Sociability indicator variance (*α_*j*[*i*]_*)	–	–	–	0.387	0.622	40.9
Within individual change over time (*δ_*t*[*i*]_*)	–	–	–	0.048	0.219	5.1
Residual (*ε_*i*_*)	–	–	–	0.298	0.546	31.6
**GxE interactions**						
G x *γ_*k*[*i*]_*	–	–	–	0.000	0.000	0.0
G x *α_*j*[*i*]_*	–	–	–	0.000	0.000	0.0
G x *δ_*t*[*i*]_*	–	–	–	0.000	0.000	0.0
**Model 4—CD38 rs1042778**						
(Intercept)	0.461	0.077	< 0.001	–	–	–
Age	−0.007	0.003	0.006	–	–	–
Gender (0 = women, 1 = men)	−0.441	0.025	< 0.001	–	–	–
rs1042778 CC-allele	−0.028	0.035	0.423	–	–	–
Assessment wave	−0.001	0.003	0.577	–	–	–
Overall between-individual variance (*γ_*k*[*i*]_*)	–	–	–	0.212	0.460	22.4
Sociability indicator variance (*α_*j*[*i*]_*)	–	–	–	0.375	0.612	39.6
Within individual change over time (*δ_*t*[*i*]_*)	–	–	–	0.045	0.212	4.8
Residual (*ε_*i*_*)	–	–	–	0.298	0.546	31.5
**GxE interactions**						
G x *γ_*k*[*i*]_*	–	–	–	0.000	0.000	0.0
G x *α_*j*[*i*]_*	–	–	–	0.014	0.117	1.4
G x *δ_*t*[*i*]_*	–	–	–	0.003	0.059	0.4

*Five sociability indicators represent overall adult sociability. In the GxE interactions, G corresponds to the fixed-effect SNP used in the model, but the interaction is taken with (G multiplies) a random-effect intercept. For example, in the Model 1, GxE interaction G x γ_k[i]_ means rs3796863 x γ_k[i]_., where γ_k[i]_ was the random-effect capturing the between-individual variance*.

**Table 6:** Individual SNPs predicting standardized social phenotypes (*n* = 1,573 participants, *n*_*y*(*i*)_ = 28,323 person-observations).

	**Sociability indicator**				
**Genotype (β [SE])**	**NEO-FFI E**	**TCI: RD1**	**TCI: RD3**	**TCI: RD4**	**EAS**
**OXTR rs1042778**					
**TT**	0.04 (0.05)	0.07 (0.03)	0.00 (0.03)	0.01 (0.03)	0.01 (0.03)
GG/GT	−0.05 (0.05)	−0.08 (0.03)	0.00 (0.03)	−0.01 (0.03)	−0.01 (0.03)
*F*-value	0.931	5.956	< 0.001	0.035	0.137
*P*-value	0.335	0.015^**^	0.990	0.853	0.712
**OXTR rs2254298**					
**GG**	0.01 (0.02)	0.01 (0.01)	0.00 (0.01)	−0.00 (0.01)	0.01 (0.01)
AA/AG	−0.05 (0.05)	−0.04 (0.03)	−0.02 (0.03)	0.02 (0.03)	−0.07 (0.03)
*F*-value	0.864	1.318	0.525	0.517	3.516
*P*-value	0.353	0.251	0.469	0.472	**0.061**^+^
**OXTR rs53576**					
**AA/AG**	0.02 (0.02)	−0.01 (0.02)	−0.00 (0.02)	0.00 (0.02)	0.00 (0.02)
GG	−0.07 (0.04)	0.02 (0.03)	0.01 (0.03)	−0.01 (0.03)	−0.01 (0.03)
*F*-value	3.481	0.819	0.120	0.120	0.152
*P*-value	**0.062**^+^	0.366	0.729	0.729	0.697
**CD38 rs3796863**					
**CC**	0.03 (0.03)	0.04 (0.02)	0.02 (0.02)	−0.01 (0.02)	−0.01 (0.02)
AA/AC	−0.05 (0.04)	−0.07 (0.03)	−0.04 (0.03)	0.01 (0.03)	0.02 (0.03)
*F*-value	1.913	7.083	2.541	0.209	0.557
*P*-value	0.167	0.008^**^	0.111	0.647	0.456

** *= p < 0.010*,

+ *= p < 0.100. NEO-FFI E = NEO-FFI Extraversion scale [df = (1, 3070)], TCI RD1, TCI RD, Sentimentality scale [df = (1, 6301)], TCI RD3, TCI RD, Social Attachment scale [df = (1, 6309)], TCI RD4, TCI RD, Dependence scale [df = (1, 6308)], EAS, EAS Sociability scale; [df = (1, 6325)]. The bolded genotype represent the alleles that have been associated with risk of social difficulties*.

## Discussion

Previous work has widely established the association between sociability and better well-being and endogenous oxytocin levels. However, due to error-proneness of personality assessments, the effects between self-reported sociability and health outcomes, or between sociability and genotype, have often been weak and inconsistent. In this study, we had two aims: first, to compare coherence and stability of five self-reported indicators for social phenotype, and second, to test whether longitudinal variance components could indirectly help to reveal GxE interactions and other reasons for inconsistencies in the literature, such as indicator heterogeneity. We did this by partitioning the population variance in adulthood sociability to trait (between-individual) variance, differences among commonly used inventories (indicator variance), state (within-individual) variance in overall sociability (i.e., the “state,” or time-variant, part of the overlapping variance of inventories) and measurement error or idiosyncratic differences that cannot be attributed to an individual, to follow up or to a sociability indicator. For the second aim, we added the “social” genotype to the model.

We found that the differences between sociability indicators contributed more to the total variance in adulthood sociability than did the trait (between-individual) variance or state (within-individual) variance. The amount of explained variance in overall adulthood sociability was ~4 times larger for between-individual variance than for within-individual variance, indicating that self-evaluated overall sociability was more a trait-like than a state-like phenomenon. Indicator variance covered two-fifths of the population variance in overall adulthood sociability. Differences in level between sociability indicators were largest in young adults (at ages 20 to 35 years) and decreased from the age 35 onwards. The sociability indicators also differed to some extent in their developmental trends: with most of the indicators, sociability decreased over the time. The exception was the sociability indicator emphasizing dependence on others' approval, which was lower among people in their twenties and increased over time to a level that corresponded to the other sociability indicators.

Regarding our second study aim, which was to test differences between sociability indicators in relation to the “social” genotype, the inclusion of genetic information to the model did not have a main effect on the within- or between-individual variance components, but it slightly decreased (explained away) the amount of sociability variance due to the differences between sociability indicators. This means that the sociability indicators might be differently associated with the genotype. When individual SNPs were used instead of the genetic risk score, the interaction between the genotype and sociability indicators variance explained in some cases even higher amount of variance in overall adulthood sociability than did the within-individual changes (state variance). Furthermore, the “sociability genes” were differently associated with different sociability indicators.

### Sociability indicator variance

Quantitative theoretical models often need to build on precise forms of “sociability.” For example, Santos et al. (2008) demonstrated that social diversity in the number and size of collaborative efforts individuals engage in could sometimes determine whether the population evolves cooperative behavior or not. Experimental animal studies also frequently make use of rather isolated forms of sociability. In contrast, studies of human psychology tend to rely on the psychometric idea that a combination of multiple imprecise indicators captures a well-defined (latent) sociability trait and reduces measurement errors in it. Despite the well-established definition of sociability as a tendency to seek and to be fond of others' company, the theoretical concept of human sociability and pragmatic solutions on how it might be best measured can be surprisingly different and error-prone in content.

Some theoretical frameworks, for example, define high sociability based on a person's popularity, their tendency to leadership, their preference to interact with others or their preference for social activities such as parties (Friedman, 2000). In other cases, the definition emphasizes the importance of social rewards (i.e., attention and acknowledgment from others), the willingness to connect on the sentimental level and to be dependent on others, or the preference for others' company instead of solitude (Buss, 1991; Cloninger et al., 1993). Differences in theoretical concepts and typical application areas also apply to the inventories used in the present study. For example, the TCI was originally developed in consideration of the underlying biological and social determinants of individual differences and is more popular in psychiatric practice and research. In comparison, the NEO-FFI is based on the individual differences that are represented in natural language and has received more attention and recognition from psychologists (De Fruyt et al., 1999; John et al., 2008). However, many authors have reported relatively small empirical differences across inventories (De Fruyt et al., 1999; Grucza and Goldberg, 2007; John et al., 2008), often proposing that some of the theoretical differences are superficial.

Whereas much of the discussion has been based on cross-sectional general personality differences, this study provides more comprehensive analysis of within- and between-individual variance in particular domain of high interest, sociability. Our findings revealed that, over time, the examined sociability-related indicators do not overlap very much in young adults, and that they have partly distinct genetic influences. More work is needed to establish psychological measures of sociability that are invariant to background conditions, and able to establish connections with theoretical predictions.

### Trait vs. state variance

Discussion on whether the personality constructs, such as sociability, should be primarily seen as states or traits has continued for decades (Steyer et al., 1999). In short, depending on the approach, the main source of the variation in sociability can be seen to be either due to differences in relatively stable personal characteristics (trait variance) or due to dynamic individual reactions to the changes in the immediate environment (state variance). Approaches based on trait variance (between-individual differences) focus on the characteristics of an individual that have great cross-situational consistency, such as differences in tendencies to react in a certain way. In contrast, approaches based on state (within-individual) variance emphasizes more both normative age-related changes (e.g., life-phase specific requirements for social behavior) and changes due to personal life events, such as crises or dysphoric mental state. Overall, the trait approach has been the dominant conceptual framework for the description of human personality (Steyer et al., 1999), but it has been questioned because temporal within-individual variations violate the personality structures derived from static between-individual differences (Molenaar and Campbell, 2009).

Even though the trait-like between-individual differences are widely recognized among psychologists regardless of their theoretical orientation, the individuals can be surprisingly inconsistent in their responses and behavior from one situation to another. For this reason, the advocates of state approach have questioned the extent to which individual's behavior can be predicted with sufficient accuracy from trait measures (Mischel, 1968; Mischel and Shoda, 2008). However, despite the possible cross-situational inconsistency of person's actual behavior or reactions, self-report questionnaires focus on individual's self-conception which, in turn, has shown to have a great, trait-like stability (McCrae and Costa, 1982). This high stability typically observed in the personality change and development literature (Roberts and DelVecchio, 2000; Bleidorn et al., 2009; Specht et al., 2011) was also supported in the present study. According to our results, a relatively small amount of variation in adulthood sociability was explained by temporal state (within-individual) variance in comparison to trait (between-individual) variance indicating that, on average, self-evaluated overall sociability is more a trait- than a state-like phenomenon.

### Gender differences and general level of development

In addition to the variance partitioning, we tested the gender differences and general level of development (an age-related mean trend) in overall adulthood sociability. Based on our results, gender differences were coherent in almost all used sociability indicators: on average, women had higher sociability than men, though the decreasing age trend was similar for both genders. Many other studies have found a similar trend regarding gender differences (Feingold, 1994; Brändström et al., 2001; Costa et al., 2001; Miettunen et al., 2007; Lippa, 2010a; Weisberg et al., 2011). It has been proposed that this could be explained by different evolutionary and sociocultural roles that men and women have, such as women on average are—or are expected to be—more nurturing, tender-minded or more “warm” and orientated toward other people than are men (Costa et al., 2001; Lippa, 2010a,b; Weisberg et al., 2011).

Despite the relatively low variation in overall adulthood sociability that was explained by state (within-individual) variance, our results indicated a significant age-related mean trend. This finding is in line with previous literature (Caspi, 1998; Roberts et al., 2001, 2006; Durbin et al., 2016). On average, people in young adulthood take on several new social roles involving commitments to relationships, work and community responsibilities. In other words, in this phase of life, the pressure on or adaptiveness of engaging in more socially active behavior can be stronger. This may partly explain why also the greater normative changes in sociability tend to take place during this time-period compared to the typically more stable life situation after the age of 30 (Durbin et al., 2016). Furthermore, our result of a stronger age effect on women suggests the possibility that external pressure for higher sociability may be stronger especially for women who are approaching the end of their reproductive years (i.e., the age of 40 years).

### The “social” genotype

Previous research has suggested that oxytocin genes might be sensitivity, or susceptibility genes for the effects of variable environment on the development of sociability and symptoms related to the social domain (e.g., Brüne, 2012; Feldman et al., 2014). That is, gene-by-environment interactions have been reported. To the extent that the relevant environment varies at the time scale we assessed, our study did not support this notion. We did not observe the implied elevations in within-individual variance in the susceptibility genotype (or any genotype thereof). However, we found that the genotype changed the variance partitioning marginally by decreasing (explaining away) variance due to differences between sociability indicators approximately by one percentage unit. In other words, differences between the sociability indicators may depend on the genetic influences. This, in turn, may partly explain the inconsistent findings in the previous literature between the endogenous oxytocin levels and the social phenotypes. In particular, we found that the genotype links with the kind of sociability that focus on sentimentality and sharing emotions with others, rather than person's preference for others' company over solitude, tendency to be attached to others or dependency on social approval. From evolutionary perspective, responsiveness to other people's emotions is a crucial element for developing capacity for social relationships and thus it has an important, adaptive function especially in the early parent-child relationships (Flanagan, 1999). Due this adaptiveness, it is logical that stronger connections are present between the genotype and the type of sociability that focus more on sentimental bonding than with other forms of sociability. This could furthermore partly explain why previous studies have reported associations especially between the sociability genes and with sensitive parenting (Feldman et al., 2012). However, due the narrow set of SNPs used in the present study, more comprehensive (e.g., genetic complex-trait or twin) studies regarding this matter are needed.

### Strengths and limitations of the study

Regarding strengths of the study, we were able to compare and study the distribution of and developmental trends in results from three inventories commonly used for measuring human sociability together with their “social” genotype in a representative, population-based study sample with over a 15-year follow-up period. Our multilevel design increased statistical power to observe associations between overall sociability and genotype relative to studies using one sample per individual. Due to the multicohort design of the Young Finns Study, we were able to examine a wide array of sociability and social preferences over most of the reproductive life span (from age 20 years or 30 years to 50 years of age depending on the inventory). The main limitations of this study are that the present data were based on self-reports, all the used inventories did not have an equal number of measurements, and despite the use of three inventories, they still represent only a subset of questionnaires widely used to assess sociability. Furthermore, the cross-national generalizability of the results remains a topic for future study, perhaps by introducing a new random effect for nationality in multinational data.

## Conclusions

We set out to study the extent to which the population variance in adulthood sociability is due to trait variance, to differences among commonly used inventories, to changes in an individual's state and to measurement error. Based on our results, different indicators for adulthood sociability are quite different in terms of development, content and how they are related to different covariates, like the “social” genotype. In addition to differences, we noted how aging decreased the differences between the sociability indicators. Although our results also indicated that self-evaluated overall sociability is more a trait- than state-like phenomenon, it may be more fruitful to avoid overly general statements on sociability, and instead concentrate on more rigorously defined sub-components and/or factors that bring together those sub-components. For example, we noted that the “sociability genes” were associated with the kind of sociability emphasizing more of sharing emotions and sentimental states of mind with others rather than more general willingness to be with others or to be depended on their company, and were not associated with the overall sociability.

## Data availability statement

These data were used under a data processing agreement in compliance with the GDPR. The raw data for this study is not publicly available, but it may be obtained by contacting the corresponding author of this article. For other requests related to the data please contact Professor Marko Elovainio (marko.elovainio@helsinki.fi) or Adjunct Professor Laura Pulkki-Råback (laura.pulkki-raback@helsinki.fi).

## Code availability

R code for analysis is provided in the Supplementary Material. R code for data processing and visualization is available upon a request from the first author (EO).

## Author contributions

LP-R, JV, TL, and OR provided the Young Finns data. EO and TR conceived the study and performed the data analysis, and EO, TR, and LK-J wrote the paper. MH and LP-R took part in writing the paper and interpreting the findings. All authors discussed the results and reviewed the final manuscript.

### Conflict of interest statement

The authors declare that the research was conducted in the absence of any commercial or financial relationships that could be construed as a potential conflict of interest.
