# Supplementary material for: A Longitudinal Multilevel Study of the “Social” Genotype and Diversity of the Phenotype
Source: Front Psychol. 2018 Oct 24;9:2034. doi: 10.3389/fpsyg.2018.02034 (PMC6207617; doi:10.3389/fpsyg.2018.02034)
Supplement: Supplementary file 1 [file Data_Sheet_1.docx]

Supplementary Material 1

**A Longitudinal Multilevel Study of**

**the “Social” Genotype and Diversity of the Phenotype**

**Elli Oksman, Tom Rosenström, Mirka Hintsanen, Laura Pulkki-Råback, Jorma Viikari, Terho Lehtimäki, Olli Raitakari and Liisa Keltikangas-Järvinen***

*** Correspondence:** Liisa Keltikangas-Järvinen: liisa.keltikangas-jarvinen@helsinki.fi

# On the model equations and random-effects design matrix

In this supplementary content, we connect our model with the more technical notation used by Douglas Bates in his book on lme4 R package. Therein, the linear mixed model is defined by two multivariate normal distributions (denoted *N*(∙ ,∙) where the first argument is mean vector and the second is covariance matrix):

$\begin{matrix} \left( Y | B=b \right) \sim N(X\beta+Zb, \sigma^{2}I) \\ B \sim N(0, \Sigma_{\theta}) \end{matrix}$ ,

where Y is a random vector modeling the n observation units, *X* is an n-by-p fixed-effects model matrix of *p* fixed covariates (including intercept), *Z* is an *n*-by-*q* random-effects model matrix, *B* is a random vector of *q* random effects and *b* its realized value, *σ^2^* is variance scaling factor, and *Σ_θ_* is a *q*-by-*q* covariance matrix of the random effects. Both *n* and *q* can be very large numbers, but *Σ_θ_* depends on only few parameters; in our case, the variance components *θ* = (*σ^2^_γ_*, *σ^2^_α_*, *σ^2^_δ_*) = (between-individual variance, sociability-indicator variance, within-individual change over time in general sociability). In practice, *Σ_θ_* is a diagonal matrix, where the first 2126 values on the diagonal correspond to *σ^2^_γ_*, the middle 6330 values to *σ^2^_α_*, and the last 10326 values to *σ^2^_δ_*. Only these three values are estimated from data. In addition to the data (including *X*), their estimated values depend on the (user-defined) random-effects model matrix, *Z*, that captures the clustering (a.k.a., dependent observations, grouping, or levels) in the data. It can be illustrative to visually examine (transpose) of *Z*, and the below supplementary figure illustrates our assigned values (the pattern of ones and zeroes respectively drawn in black and white).


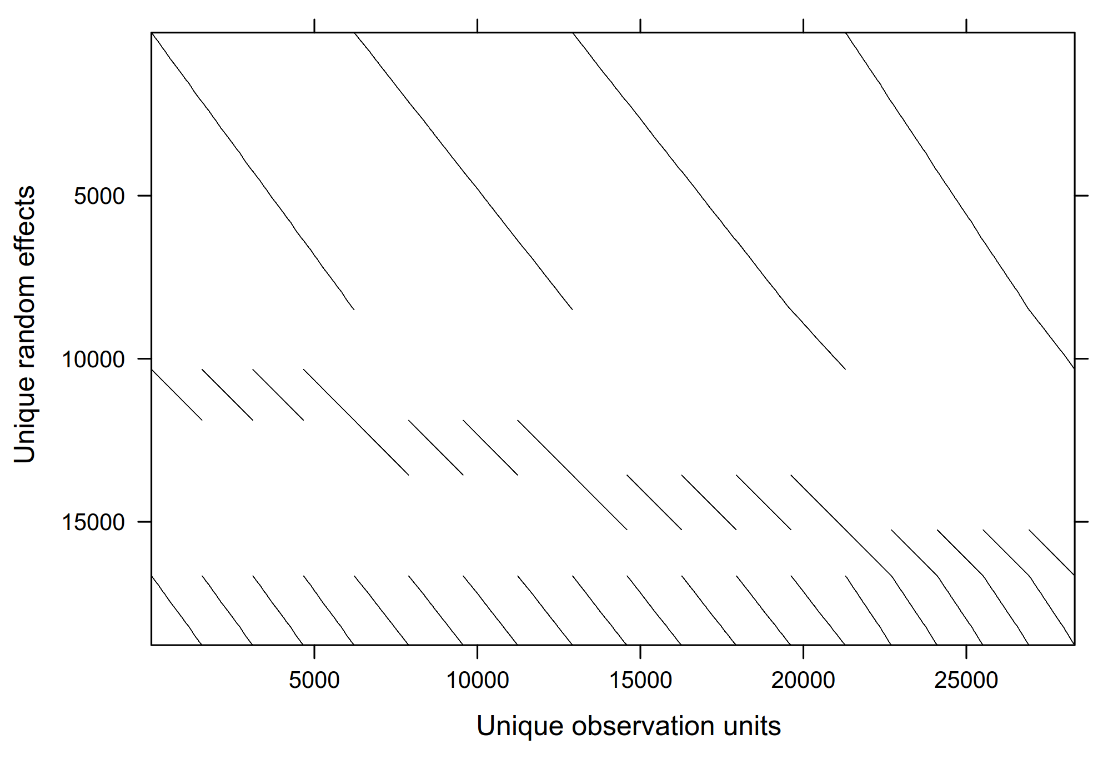


In this figure, *y*-axis corresponds to unique random effects and *x*-axis to unique observation units. The black points in the image signify the occurrences of the same random effect in multiple unique units. The ‘lower’ 2126 random-effects occur as many times as there are observations from the same individual, are independent of each other, and have the variance *σ^2^_γ_*. They capture individual specific level of the outcome units. The next 6330 random effects occur only four times for the four distinct time points given sociability indicator was assessed from given individual. The remaining (upper) 10326 random effects instead occur four times for the four sociability indicators assessed in given time point from given individual. Now, all the random effects in the vector *B* are independent of each other according to the model, but their best-fitting variance estimates according to REML estimate in observed data heavily depend on our definition of *Z*. As the random effects with the variance *σ^2^_γ_* always stay constant for given individual, their variance must correspond the parts of the data that vary strictly between individuals. The random effects with variance *σ^2^_α_* also vary between individuals, but in addition, across the time points they were studied. They stay constant across the indicators they were studied with in given time. From this it might seem that both *σ^2^_γ_* and *σ^2^_α_* could reflect individual-specific effects to a varying degree, but then the model would necessarily fit data worse than when these effects are left to those random effects that can only model them. Thus, *σ^2^_γ_* ends up modeling primarily general between-individual effects and *σ^2^_α_* ends up modeling primarily the variance of indicators across the time points. An analogous logic applies to *σ^2^_δ_*.

The above illustration describes how we captured our clustered data structure into different variance components. In addition, there is a general residual-variance component for random effects that are unique to each observation. As the usual logic goes, and the logic in above, this variance component represents everything not explained by the other random effects and the fixed effects (note that also the above variance components accommodate to fixed effects in order to best fit the data; i.e., are reduced by addition of fixed covariates). We encourage readers interested in more details to search the web for Douglas M. Bates’ freely available book “lme4: Mixed-effects modeling with R” (currently available, e.g., in URLs http://lme4.r-forge.r-project.org/book/ and http://webcom.upmf-grenoble.fr/LIP/Perso/DMuller/M2R/R_et_Mixed/documents/Bates-book.pdf).
