# Supplementary material for: A Longitudinal Multilevel Study of the “Social” Genotype and Diversity of the Phenotype
Source: Front Psychol. 2018 Oct 24;9:2034. doi: 10.3389/fpsyg.2018.02034 (PMC6207617; doi:10.3389/fpsyg.2018.02034)
Supplement: Supplementary file 2 [file Data_Sheet_2.docx]

Supplementary Material 2

**A Longitudinal Multilevel Study of**

**the “Social” Genotype and Diversity of the Phenotype**

**Elli Oksman, Tom Rosenström, Mirka Hintsanen, Laura Pulkki-Råback, Jorma Viikari, Terho Lehtimäki, Olli Raitakari and Liisa Keltikangas-Järvinen***

*** Correspondence:** Liisa Keltikangas-Järvinen: liisa.keltikangas-jarvinen@helsinki.fi

# R code

# sos = overall adulthood sociability (five sociability indicators combined, *y_i_*)

# m.id = within individual change over time (*δ_t[i]_*)

# v.id = sociability indicator variance (*α_j[i]_*)

# id = overall between-individual variance (*γ_k[i]_*)

# Assessment wave

dd$time[dd$time == "1"] <- 0 #1997

dd$time[dd$time == "2"] <- 4 #2001

dd$time[dd$time == "3"] <- 10 #2007

dd$time[dd$time == "4"] <- 15 #2012

# Table 3

# Model 1:

library(lme4) # load the mixed-effect modeling tools (lmer function)

M1 <- lmer(sos ~ age + gender + time + (1|m.id) + (1|v.id) + (1|id), data = d)

# Model 2 (Genetic risk score and GxE interactions included to Model 1):

M2 <- lmer(sos ~ age + gender + genrisk + time + (1 | m.id) + (1 | v.id) + (1 | id) +

(0 + genrisk | m.id) + (0 + genrisk | v.id) + (0 + genrisk | id), data=d)

# Table 4:

# Individual SNPs and GxE interactions predicting standardized overall adulthood sociability

# Model 1 - OXTR rs3796863

M1snp <- lmer(sos ~ age + gender + rs3796863A + time + (1 | m.id) + (1 | v.id) +

(1 | id) + (0 + rs3796863A | m.id) + (0 + rs3796863A | v.id) +

(0 + rs3796863A | id), data=d)

# Model 2 - OXTR rs2254298

M2snp <- lmer(sos ~ age + gender + rs2254298A + time + (1 | m.id) + (1 | v.id) +

(1 | id) + (0 + rs2254298A | m.id) + (0 + rs2254298A | v.id) +

(0 + rs2254298A | id), data=d)

# Model 3 - OXTR rs53576

M3snp <- lmer(sos ~ age + gender + rs53576A + time + (1 | m.id) + (1 | v.id) + (1 | id) +

(0 + rs53576A | m.id) + (0 + rs53576A | v.id) + (0 + rs53576A | id),

data=d)

# Model 4 - CD38 rs1042778

M4snp <- lmer(sos ~ age + gender + rs1042778G + time + (1 | m.id) + (1 | v.id) +

(1 | id) + (0 + rs1042778G | m.id) + (0 + rs1042778G | v.id) +

(0 + rs1042778G | id), data=d)

# Table 5:

# Individual SNPs predicting standardized social phenotypes

# rs1042778G

summary(lm(NEO ~ rs1042778G, data=d)) # NEO-FFI Extraversion

summary(lm(RD1 ~ rs1042778G, data=d)) # TCI: RD1 Sentimentality

summary(lm(RD3 ~ rs1042778G, data=d)) # TCI: RD3 Social attachment

summary(lm(RD4 ~ rs1042778G, data=d)) # TCI: RD4 Dependence

summary(lm(EAS ~ rs1042778G, data=d)) # EAS Sociability

# rs2254298A

summary(lm(NEO ~ rs2254298A, data=d))

summary(lm(RD1 ~ rs2254298A, data=d))

summary(lm(RD3 ~ rs2254298A, data=d))

summary(lm(RD4 ~ rs2254298A, data=d))

summary(lm(EAS ~ rs2254298A, data=d))

# rs53576A

summary(lm(NEO ~ rs53576A, data=d))

summary(lm(RD1 ~ rs53576A, data=d))

summary(lm(RD3 ~ rs53576A, data=d))

summary(lm(RD4 ~ rs53576A, data=d))

summary(lm(EAS ~ rs53576A, data=d))

# rs3796863A

summary(lm(NEO ~ rs3796863A, data=d))

summary(lm(RD1 ~ rs3796863A, data=d))

summary(lm(RD3 ~ rs3796863A, data=d))

summary(lm(RD4 ~ rs3796863A, data=d))

summary(lm(EAS ~ rs3796863A, data=d))
